# COVID-19-Related Rhabdomyolysis: A Case Report and Literature Review

**DOI:** 10.7759/cureus.97300

**Published:** 2025-11-20

**Authors:** Apurva Ashok, Alifya A Lokhandwala, Seohyeon Im, Rohit Sharma, Daniela Urma

**Affiliations:** 1 Internal Medicine, Mass General Brigham Salem Hospital, Salem, USA

**Keywords:** acute kidney failure, covid-19, creatine kinase, non-traumatic rhabdomyolysis, viral-induced rhabdomyolysis

## Abstract

Rhabdomyolysis is the release of myoglobin, creatine kinase (CK) and potassium from damaged skeletal muscle into the bloodstream. This can result in life-threatening complications such as arrhythmias, kidney failure, disseminated intravascular coagulation (DIC) and cardiac arrest. The risk of developing rhabdomyolysis in COVID-19 has been published in a handful of case reports and series, although most of the patients in these studies have a strong comorbid history. We present a healthy 21-year-old male patient who presented with CK levels of >450,000 U/L two weeks after non-specific flu-like symptoms, in the absence of a second triggering event, but was found to be COVID-positive. In addition, the cardiac-specific troponins and liver function tests were deranged, which prompted investigations for hepatitis and myocarditis. Broad infectious work-up and imaging studies were negative, and the patient’s laboratory derangements were significantly improved >70% after seven days of hospital admission. We also highlight the current literature available about this topic, mainly limited to case reports.

## Introduction

Rhabdomyolysis is characterized by the breakdown of skeletal muscle myocytes when induced by traumatic stressors such as burns, excessive exertion, and crush injuries or non-traumatic causes including metabolic derangements, seizures, infections, and drugs [1]. The common symptoms and signs include muscle pain and tenderness, dark-colored urine due to myoglobinuria, and elevated creatine kinase levels (typically greater than 1000 IU/L) [1]. However, this disease has a spectrum that causes severe, life-threatening complications such as cardiac dysrhythmias, acute tubular necrosis (ATN), disseminated intravascular coagulation (DIC), compartment syndrome and possibly death. 

Viral infections such as influenza and enterovirus are recognized triggers of rhabdomyolysis due to direct viral invasion into the myocyte and the body's own immune response, leading to muscle inflammation. The exact incidence and mechanism of rhabdomyolysis following COVID-19 remains unclear, with only a few case reports in older patients and retrospective studies documenting this association so far [2-4]. This case emphasizes its continued association, particularly in a young patient without pre-existing co-morbidities or risk factors, and highlights its potential to affect multiple organ systems. 

## Case presentation

A 21-year-old healthy Caucasian man presented to the emergency department (ED) with a two-week history of upper respiratory symptoms, generalized malaise, weakness, and a one-day history of fever, cough, and chills. He had no notable past medical history and was a college student. The patient self-administered a COVID-19 antigen at-home test, which was negative. Approximately one week after the onset of symptoms, he experienced diffuse muscle aches in his thighs and back and was unable to perform his regular exercise. The patient started noticing cola-colored urine, which eventually led him to present to the hospital. Throughout this time, the patient denied any chest pain, shortness of breath, abdominal pain, dysuria or vomiting. He denied any strenuous exercise or activity, recreational drug use, trauma, crush or burn injuries, use of restraints or tick bites. There was no evidence of environmental heat illness, carbon monoxide exposure or metabolic myopathies.

On presentation to the ED, the patient’s blood pressure was 175/104 with a regular heart rate and rhythm. He was afebrile, and oxygen saturation was 99% on room air. The physical exam was positive for bilateral tenderness to palpation in the thighs, normal strength in upper and lower extremities, and the absence of peripheral edema. The remainder of the cardiovascular, respiratory, and abdominal examination was within normal limits. 

The patient’s COVID-19 polymerase chain reaction (PCR) test was positive on admission, and the creatinine kinase (CK) was elevated at 490,440 U/L. The comprehensive metabolic panel revealed elevated creatinine, aspartate aminotransferase (AST), alanine aminotransferase (ALT) and normal alkaline phosphatase (ALP) and total bilirubin. The troponin T was elevated at 1,195 ng/L with a normal NT-pro BNP. The lactate dehydrogenase (LDH) level was elevated, and the electrolytes and white blood cell count were within reference ranges. He was admitted to the hospital for fluid resuscitation and further evaluation of the acute kidney injury (classified Stage 2 based on his baseline creatinine of 0.6 mg/dL), as well as troponin and transaminase elevation (Table 1).

**Table 1:** Quantitative labs from day zero (admission day) to day six (day of discharge) CK: creatine kinase; RR: reference range; AST: aspartate aminotransferase; ALT: alanine aminotransferase; ALP: alkaline phosphatase; HS: high sensitivity; NA: not available

Lab tests	Reference range	Day 0	Day 1	Day 2	Day 3	Day 4	Day 5	Day 6
CK (U/L)	47-322	490,440	328,820	163,160	53,780	47,100	13,858	8,034
Creatinine (mg/dL)	0.60-1.30	1.20	1.38	1.29	1.20	1.09	1.21	1.18
AST (U/L)	15-41	2,540	1,724	1,116	693	501	324	NA
ALT (U/L)	10-50	514	452	355	388	480	451	NA
ALP (U/L)	40-130	42	35	30	39	39	39	NA
Troponin T-HS Gen 5 (ng/L)	0-9	1,195	1,245	NA	NA	NA	NA	NA

Further testing for the elevated transaminases included HIV and hepatitis A, B and C panel, and Epstein-Barr Virus (EBV) and Cytomegalovirus (CMV) screening as causes for viral hepatitis. The urine toxicology and viral flu panel were negative. An ultrasound of the liver revealed no evidence of a localized intrinsic process and demonstrated normal portal vein patency and flow, hence we excluded malignancy, portal vein thrombosis, and cholecystitis. The elevated cardiac enzymes were further investigated with an echocardiogram to assess for myocarditis, which demonstrated normal filling pressures and preserved bi-ventricular function. An electrocardiogram (EKG) revealed sinus rhythm, an incomplete right bundle branch block (RBBB), and no ischemic changes that were concerning for a myocardial infarction. 

In terms of management, the patient received adequate fluid resuscitation with four liters of normal saline in the emergency department. He was later maintained on additional lactate ringers' solution at 150-250 ml per hour, to complete an additional five liters on day two, 1350 ml on day three and 1000 ml on day four. His initial cola-colored urine normalized over the first three days. Due to adequate urine output and rapid decline of the CK, he did not require dialysis. Additionally, he was started on nirmatrelvir/ritonavir (Paxlovid) on the second day of admission for two doses only due to concern over the medication’s effect on the liver. Remdesivir was also contraindicated due to the deranged liver function tests (LFTs) and the patient did not require dexamethasone as his saturation was maintained and he did not report any respiratory concerns. Pulse-dosed steroids were initially considered for any underlying inflammation or infection, however due to the patient’s unremarkable vitals and benign physical examination, steroids were not given. The patient did not develop any new symptoms and reported significant improvement but not complete resolution of his myalgias, weakness, and myoglobinuria over the course of his seven-day admission. Due to his improvement in symptoms, other causes of myalgia, such as inflammatory or autoimmune causes, were not investigated. The patient was followed for a year through chart review without any further complications from this disease. Table 1 displays the progression of the patient’s laboratory results throughout the hospital course. 

## Discussion

We report a young man with no comorbidities presenting with acute rhabdomyolysis approximately one week after COVID-19 infection, with an elevated CK of 450,000 U/L, acute kidney injury (AKI), deranged transaminases and elevated cardiac troponins. His AKI was likely secondary to ATN, which is found in up to 15% of patients and is caused by the direct toxicity of myoglobin to the renal tubular cells, as well as the formation of intratubular casts [5]. His elevated troponin and LFT derangement were likely due to cardiac myocyte and hepatocyte cell lysis; there was also concern for myocarditis and hepatitis, as these have been associated with COVID-19 [6]. Although we did not see other complications such as compartment syndrome, DIC or cardiac dysrhythmias, this case highlights the multi-system impact of the disease, even in the absence of significant comorbidities. 

The current literature has documented case reports and retrospective observational studies which highlight older patients with more comorbidities, such as diabetes, chronic kidney disease and hypertension, hence increasing their risk of presenting with worse signs and symptoms of rhabdomyolysis [1]. These studies have been reported in Table 2 [7-10].

**Table 2:** Literature review of previous case reports and studies about COVID-associated rhabdomyolysis CK: creatine kinase

Study/Case report and type	Population/Details	Key findings
Case report [7]	64-year-old woman with myalgia, fatigue, and significantly elevated CK levels of 119,000 U/L	Fully recovered after intravenous fluid support without residual renal impairment.
Histopathological analysis [8]	Post-mortem examination of the kidneys of patients with COVID-19	Pigmented casts in 3 cases, suggesting rhabdomyolysis possibly due to COVID treatments or hyperventilation.
Case report [9]	16-year-old boy with COVID-19-associated rhabdomyolysis. Highest CK Level Recorded (427,656 U/L)	His initial CK level was 427,656 U/L, while his serum creatinine remained normal for his age on admission and throughout the hospital stay.
Case report [10]	68-year-old male with symptomatic COVID-19 pneumonia and traumatic injuries. High CK level in adults (146,328 U/L)	Presented with symptomatic COVID-19 pneumonia and traumatic injuries, including rib fractures and a rectus sheath hematoma, which contributed to the development of severe rhabdomyolysis

The highest recorded COVID-associated CK was 427,656 U/L in a 16-year-old male patient with previous episodes of viral illness-induced rhabdomyolysis [9]. Additionally, as per a recent case report published in 2022, a 64-year-old woman developed COVID-related rhabdomyolysis after presenting with myalgia, fatigue, and significantly elevated CK levels. After receiving intravenous fluid support, she fully recovered without any residual renal impairment [7]. 

Early studies during the pandemic indicated that rhabdomyolysis following COVID-19 illness was linked to a higher risk of complications, ICU admissions, and increased in-hospital mortality [11]. Furthermore, a recent histopathological analysis examining post-mortem COVID-19 patients’ kidneys revealed pigmented casts in three out of 26 cases, possibly indicating rhabdomyolysis secondary to COVID treatments or COVID-related hyperventilation. The authors of the study did not exclude the possibility of direct virus-induced muscle damage [8]. Concurrently, rhabdomyolysis has also been linked with other viral and bacterial infections, such as influenza, severe acute respiratory syndrome (SARS), streptococcus, and legionella [12]. 

COVID-19 can also be linked to other organ dysfunction, apart from rhabdomyolysis. These include myocarditis and hepatitis. COVID-19-related myocarditis is caused by the virus directly affecting myocytes, immune system-related damage, and injury to small blood vessels. According to the American College of Cardiology, myocarditis in COVID-19 patients may stem from an overactive immune response, marked by high levels of cytokines and impaired endothelial function [13]. Myocarditis is relatively uncommon among hospitalized COVID-19 patients but is associated with a markedly higher risk of poor outcomes. These include increased in-hospital mortality and complications such as cardiogenic shock and AKI [13]. Rhabdomyolysis may also be associated with noncardiac elevations of high-sensitivity troponin related to troponin T isoform expression in diseased/damaged skeletal muscle, which is most likely what we see in our patient. Furthermore, COVID-19-related hepatitis can be characterized by damage to the hepatocytes and cholangiocytes through exaggerated immune-mediated responses, hypoxia, and systemic inflammatory reactions. In addition, the virus increases susceptibility to liver injury and damage by binding to angiotensin-converting enzyme 2 which is expressed in hepatocytes [11]. This is particularly detrimental in patients with pre-existing liver disease such as non-alcoholic fatty liver disease or chronic viral hepatitis. 

## Conclusions

In summary, this case of a young patient with no past comorbidities adds to the growing evidence of COVID-19’s potential to cause severe extra-pulmonary manifestations, emphasizing the need for continued vigilance in patients without risk factors and continued research in understanding its pathophysiology. Vigilance must be maintained in keeping myocarditis and hepatitis in the differential diagnoses. Early recognition and management of rhabdomyolysis, including addressing potential renal, hepatic, and cardiac complications, are crucial for favorable outcomes. Lastly, timely and adequate fluid resuscitation can minimize the risk of long-term damage caused by acute tubular necrosis and hepatocellular injury. Although this is a single case report, there is scope to consider further studies to analyze the true causation between COVID-19/other viral illnesses and rhabdomyolysis. 

## References

[1] Torres PA, Helmstetter JA, Kaye AM, Kaye AD (2015). Rhabdomyolysis: pathogenesis, diagnosis, and treatment. Ochsner J.

[2] Jin M, Tong Q (2020). Rhabdomyolysis as potential late complication associated with COVID-19. Emerg Infect Dis.

[3] Solís JG, Esquivel Pineda A, Alberti Minutti P, Albarrán Sánchez A (2020). Case report: rhabdomyolysis in a patient with COVID-19: a proposed diagnostic-therapeutic algorithm. Am J Trop Med Hyg.

[4] Farouji A, Hellou R, Peretz A (2021). Asymptomatic rhabdomyolysis in a young adult with COVID-19. Cureus.

[5] Runnstrom M, Ebied AM, Khoury AP, Reddy R (2018). Influenza-induced rhabdomyolysis. BMJ Case Rep.

[6] De Rosa A, Verrengia EP, Merlo I, Rea F, Siciliano G, Corrao G, Prelle A (2021). Muscle manifestations and CK levels in COVID infection: results of a large cohort of patients inside a pandemic COVID-19 area. Acta Myol.

[7] Bawor M, Sairam S, Rozewicz R, Viegas S, Comninos AN, Abbara A (2022). Rhabdomyolysis after COVID-19 infection: a case report and review of the literature. Viruses.

[8] Su H, Yang M, Wan C (2020). Renal histopathological analysis of 26 postmortem findings of patients with COVID-19 in China. Kidney Int.

[9] Anwar H, Al Lawati A (2020). Adolescent COVID-19-associated fatal rhabdomyolysis. J Prim Care Community Health.

[10] Riccardi J, Fredericks CJ, Callcut RA (2022). Trauma and COVID-induced severe rhabdomyolysis. Am Surg.

[11] Haroun MW, Dieiev V, Kang J (2021). Rhabdomyolysis in COVID-19 patients: a retrospective observational study. Cureus.

[12] Xu Z, Shi L, Wang Y (2020). Pathological findings of COVID-19 associated with acute respiratory distress syndrome. Lancet Respir Med.

[13] Bradley BT, Maioli H, Johnston R (2020). Histopathology and ultrastructural findings of fatal COVID-19 infections in Washington State: a case series. Lancet.

